# Efficacy and Feasibility of the Submuscular Implantation Technique for an Implantable Cardiac Electrical Device

**Published:** 2014-10-30

**Authors:** Shinichi Asamura, Takashi Kurita, Koichiro Motoki, Ryobun Yasuoka, Takahiro Hashimoto, Noritaka Isogai

**Affiliations:** ^a^Departments of Plastic and Reconstructive Surgery, Kinki University Faculty of Medicine. Osaka-Sayama, Osaka, Japan; ^b^Departments of Cardiology, Kinki University Faculty of Medicine. Osaka-Sayama, Osaka, Japan

**Keywords:** implantable cardiac electrical devices, pectoralis major muscle, skin-related complications, cardiologist, Asians

## Abstract

**Background**: A common complication associated with implantable cardiac electrical device implantation compromises skin lesions caused by overstretching just above a buried device that is relatively large in size. Apart from affecting the cosmetic appearance in some patients, a compromised blood supply to the skin may also lead to ischemic necrosis, which is an important complication. We describe a novel procedure for the implantation of implantable cardiac electrical devices generators under the pectoralis major muscle to avoid such skin-related complications. **Methods**: Twenty-one patients were referred to plastic surgeon for surgical support for the secondary replacement of implantable cardiac electrical devices. In all cases, the leads and devices had been implanted under the skin. We decided to perform device implantation under the pectoralis major muscle, which was highly recommended in all these patients. **Results**: In Japan, leanness is determined on the basis of body mass index less than 18.5, and 11 patients out of 21 (52%) were considered to be lean. The surgeon's participation in the procedure for implantable cardiac electrical device implantation did not exceed 5 minutes in total.

**Conclusions:** We consider that the novel method of sub–pectoralis major muscle device implantation described here minimizes the risk of the skin breakdown and improves the patient's quality of life.

The implantable cardiac electrical devices (ICEDs) are commonly used, that is, pacemaker and implantable cardioverter-defibrillator (ICD). These consist of a pulse generator and 1 or more electrically active lead(s) and are usually implanted transvenously by cardiologists. Most of pacemaker systems are buried in the subclavian area, and the operator needs to pay careful attention to creating an appropriately sized subcutaneous pocket that is mainly based on the volume of the pulse generator.^1^^,^^2^ Although a considerable decrease in the size of ICED has been achieved over time, the size of an ICD generator is still 4 times larger than that of a standard pacemaker.^3^

Over the last 3 decades, ICED technology has progressed considerably and has been proven to improve the quality of life and life expectancy in patients of arrhythmic disorders.^4^^-^6 The number of newly implanted ICEDs has been increasing because of an increasingly aging population and the adoption of more aggressive indication criteria; moreover, this trend may also lead to an increase in the number of periodical generator replacements. With this increase in the number of surgical ICED implantations, a higher incidence of complications related to ICED implantation is an important concern.^7^^,^^8^

A common complication associated with ICED implantation compromises skin circulation caused by overstretching just above a buried device that is relatively large in size.^7^ Apart from affecting the cosmetic appearance in some patients, a compromised blood supply to the skin may also lead to ischemic necrosis, which is an important complication.

To resolve this issue, a new procedure or technique has been needed. In this report, we describe a novel procedure for the implantation of ICED generators under the pectoralis major muscle (PMM) to avoid such skin-related complications.

## MATERIALS AND METHODS

### Patients

**Figure 1:**
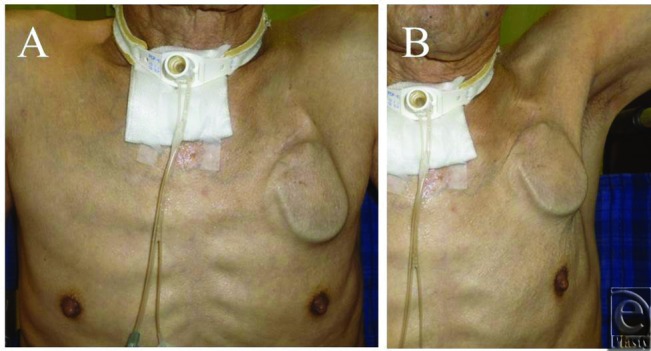
The ICD was implanted under the skin. (*a*) Upright position. The ICD looks protruding from the chest wall. (*b*) When he shoulder was elevated, the upper margin of the ICD is moved to the skin incision.

From October 2007 to February 2012, all 21 patients (all men) were referred from the cardiologist at our institution. They inserted the initial ICED under the skin, and for the purpose of the second replacement of the ICED, they preferred plastic surgeon to take care of this part of the treatment simply, because they are not surgical-oriented specialists. The indication for device replacement was battery depletion and overstretched skin except for 2 patients in whom skin breakdown probably due to ischemic skin necrosis or infection was observed prior to battery depletion (Fig 1). The body mass index (BMI: mass [kg]/height^2^ [m]) of all the patients was calculated.^9^

We decided to perform device implantation under the PMM, which was highly recommended in all these patients. The preexisting leads had been inserted through the subclavian vein and the number and the location of the leads were determined on the basis of the primary cardiac disease of each patient. Lead replacement was not required in any of the patients.

The role of the plastic surgeon was to create a tissue pocket for the implantation of the ICED below the PMM with a cardiologist. We describe the technique for creating such a pocket for ICED implantation.

### Surgical technique

**Figure 2:**
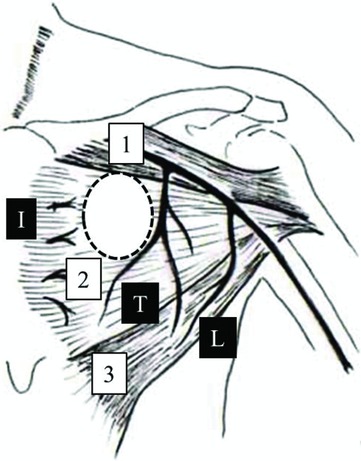
Anatomy of the PMM. The dotted-lined circle indicates the pocket where the device must be implanted: (1) Clavicular segment, (2) Sternocostal segment, and (3) Abdominal segment; (T) Thoracoacromial artery, (L) Lateral thoracic artery, and (I) Internal thoracic artery.

The procedure was performed under local anesthesia. A skin incision was made below the left clavicle along the previous surgery scar, deep enough to reach the surface of the fascia of the PMM medial to the thoracoacrominal vessel. Therefore, the risk of accidental injury with the skin incision is nonexistent. The fascial incision was made with an electric knife along the lower margin of the clavicular segment. The PMM was manually separated between the clavicular and sternocostal segments, and the second rib was exposed. The loose connective tissue under the PMM was removed manually, extending the incision if necessary until the fourth rib was well visualized. With these surgical maneuvers, an adequate space was created for the device to be implanted (Fig 2). The rest of the procedure is usually handled by the cardiologist, for example, placement of the device and lead hook-up.

## RESULTS

In Japan, leanness is determined on the basis of BMI less than 18.5 and 11 patients (11/21; 52%) were considered to be lean.

The surgeon's participation in the procedure for ICED implantation did not exceed 5 minutes in total. The amount of bleeding was negligible in all patients.

**Figure 3:**
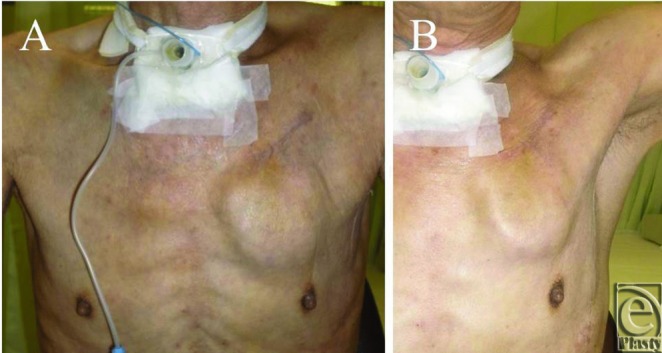
The ICD was implanted under the PMM. (*a*) Upright position. The bulging is still the ICD present but slightly more natural than directly under the skin. (*b*) When he shoulder was elevated, the ICD remains at the same position.

The mean follow-up period was 25 months (range: 14–38 months). During the postoperative follow-up period, we were prepared for unexpected complications following this new procedure; however, there have been no significant complications thus far (Fig 3). Not even a single case of pocket erosion, infection, hematoma, or seroma was recorded during the entire follow-up.

## DISCUSSION

ICED implantation is a frequently used procedure for improving the quality of life and life expectancy of patients with arrhythmia.^1^^-^8 With the prolongation of the patient's life, more frequent device replacement may be required, possibly leading to a higher risk of complications.

With comparatively lower BMIs, Asian individuals are likely to have thinner subcutaneous fatty tissues than people of Caucasian origin and other ethnicities. In fact, more than 50% of our patients had a BMI of less than 18.5. In such patients, implantation of the device under the PMM would be more natural from the cosmetic perspective, with a possibly improved perception of device stability.

Before the device replacement procedure, most patients complained about accidental movement of the device during daily activities or rubbing the skin over the device when bathing (Fig 1). However, the deeper placement under the PMM allows for lesser protrusion of the device as compared to normal subcutaneous placement; follow-up examinations in our patients revealed patients to be more comfortable with the new placement of the device (Fig 3). So far, we do not have to remove the device because of any complication on the rib bone of periosteum. The risk of long-term complications due to the device placement seems to be very low if any.

The advantage of our procedure is that there is only 1 skin incision; in addition, with the lateral approach, the device can be loosened and can be moved laterally; therefore, it is firmly pressed to stabilize it with the wide adhesive tape after the replacement for a few weeks. Our approach does not require stabilizing at all or drainage from incision.^10^^,^^11^

We recognized that deeper implantation of the device, that is, under the PMM, may result in the need for more invasive procedures, possibility causing greater bleeding and longer operative times. However, in this study, no such adverse issues were noted during this novel procedure, which was performed by cardiologists.

To minimize the surgical invasion, accurate knowledge of the anatomy of PMM is essential. The PMM consist of 3 major segments as follows: a clavicular segment arising from the medial half of the clavicle, a sternocostal segment originating from the sternum and the anterior surface of the upper 6 costal cartilages, and the abdominal segment arising from the anterior rectus sheath. These 3 segments merge and are inserted into the crista tuberculi majoris of the upper humerus.^12^ These segments are loosely attached to each other and are visually recognizable (Fig 2). In our experience, the clavicular and sternocostal segments can be manually separated without any significant bleeding.

The primary blood supply to the PMM is through the thoracoacromial artery, with minor contributions from other vessels, such as the lateral thoracic artery and the internal thoracic artery. It is important that the skin incision should be made medial to the thoracoacromial artery.^13^ Blunt dissection between the clavicular and sternocostal segments can cause significant bleeding unless meticulous attention is paid to the location of the thoracoacromial artery (Fig 2).

All 21 patients had undergone the initial subcutaneous ICED implantation by cardiologists, and they experienced skin problems over the site of generator. Typically, cardiologists tend to be cautious with respect to the risk of bleeding complications, particularly when muscle tissue is severed. In reality, as long as cardiologists are aware of the location of the thoracoacromial artery, the procedure for creating a sub-PMM pocket is fairly easy and safe.

## CONCLUSIONS

It does not require any specialized surgical skills; hence, even cardiologists with relatively little surgical experience can perform this procedure. On the basis of our experience, we consider that the novel method of sub-PMM device implantation described here minimizes the risk of the skin breakdown and improves the patient's quality of life. We strongly recommend that such devices be implanted under the PMM, even for initial implantation.
